# Dynamics of 5-methylcytosine and 5-hydroxymethylcytosine during pronuclear development in equine zygotes produced by ICSI

**DOI:** 10.1186/s13072-017-0120-x

**Published:** 2017-03-15

**Authors:** Sonia Heras, Katrien Smits, Catharina De Schauwer, Ann Van Soom

**Affiliations:** Department of Reproduction, Obstetrics and Herd Health, Faculty of Veterinary Medicine, Ghent University, 9820 Merelbeke, Belgium

**Keywords:** Horse, Pronucleus, Epigenetic reprogramming, 5-Methylcytosine, 5-Hydroxymethylcytosine, DNA methylation, DNA hydroxymethylation, Active demethylation

## Abstract

**Background:**

Global epigenetic reprogramming is considered to be essential during embryo development to establish totipotency. In the classic model first described in the mouse, the genome-wide DNA demethylation is asymmetric between the paternal and the maternal genome. The paternal genome undergoes ten-eleven translocation (TET)-mediated active DNA demethylation, which is completed before the end of the first cell cycle. Since TET enzymes oxidize 5-methylcytosine to 5-hydroxymethylcytosine, the latter is postulated to be an intermediate stage toward DNA demethylation. The maternal genome, on the other hand, is protected from active demethylation and undergoes replication-dependent DNA demethylation. However, several species do not show the asymmetric DNA demethylation process described in this classic model, since 5-methylcytosine and 5-hydroxymethylcytosine are present during the first cell cycle in both parental genomes. In this study, global changes in the levels of 5-methylcytosine and 5-hydroxymethylcytosine throughout pronuclear development in equine zygotes produced in vitro were assessed using immunofluorescent staining.

**Results:**

We were able to show that 5-methylcytosine and 5-hydroxymethylcytosine both were explicitly present throughout pronuclear development, with similar intensity levels in both parental genomes, in equine zygotes produced by ICSI. The localization patterns of 5-methylcytosine and 5-hydroxymethylcytosine, however, were different, with 5-hydroxymethylcytosine homogeneously distributed in the DNA, while 5-methylcytosine tended to be clustered in certain regions. Fluorescence quantification showed increased 5-methylcytosine levels in the maternal genome from PN1 to PN2, while no differences were found in PN3 and PN4. No differences were observed in the paternal genome. Normalized levels of 5-hydroxymethylcytosine were preserved throughout all pronuclear stages in both parental genomes.

**Conclusions:**

In conclusion, the horse does not seem to follow the classic model of asymmetric demethylation as no evidence of global DNA demethylation of the paternal pronucleus during the first cell cycle was demonstrated. Instead, both parental genomes displayed sustained and similar levels of methylation and hydroxymethylation throughout pronuclear development.

## Background

During mammalian development, two major waves of epigenetic reprogramming take place, one during germ line differentiation and the second during preimplantation embryo development. The epigenetic reprogramming during embryo development is of major importance because it is considered as being essential to establish a totipotent state [1]. The methylation of the fifth carbon of cytosine, 5-methylcytosine (5mC), was the first epigenetic modification discovered in DNA and, hence, is nowadays the best studied DNA epigenetic modification. It plays a key role in gene expression regulation, X chromosome inactivation, gene imprinting and the control of endogenous retrotransposons [2]. Based on studies in the mouse, the genome-wide DNA demethylation during embryo development, which excludes imprinted genes and retrotransposons, is proposed to be asymmetric between the maternal and the paternal genome [3, 4]. As such, the complete demethylation of the paternal DNA is achieved before the end of the first cell cycle by active demethylation. In contrast, the demethylation of the maternal genome is a passive process associated with cell divisions [5]. This epigenetic reprogramming during preimplantation embryo development, however, is not strictly conserved between species. While this asymmetric global demethylation pattern is conserved in rat [2, 6] and human [7], some species such as rabbit [8], pig [9], goat [10] and sheep [11] failed to follow this model. In these species, no DNA demethylation is observed during the first cell cycle, regardless of the parental origin of the genome. Other species, such as cattle, show an intermediate pattern with partial demethylation of the paternal pronucleus (pPN) in the zygote [11].

In 2009, a new modified form of cytosine, 5-hydroxymethylcytosine (5hmC), was identified. This new modification is generated by the oxidation of 5mC by the ten-eleven translocation (TET) enzymes [12, 13], which are able to further oxidize 5hmC into 5-formylcytosine (5fC) and 5-carboxylcytosine (5caC) [12]. As such, TET3 is considered to be the initiator of active DNA demethylation in the paternal genome. The protein STELLA (PGC7), on the other hand, protects the maternal genome from active demethylation by binding to the di-methylated histone H3 lysine 9 (H3K9me2) and excluding TET3 which results in preventing the oxidation of 5mC to 5hmC [14]. The fact that 5hmC was considered then a DNA demethylation transient, was supported by the observed complementary patterns of 5mC and 5hmC in the pPN during pronuclear development in mouse, rabbit and cattle, in which a reduction in 5mC levels is accompanied by an increase in 5hmC levels [15]. Though, the presence of high 5hmC levels in several tissues, including the nervous system, indicates that this epigenetic DNA modification plays its own epigenetic role [13] being involved in chromatin and transcription regulation [16, 17].

The biological implication of this lack of interspecies conservation of global asymmetric DNA demethylation is currently unknown. Moreover, the contradictory results observed within the same species using different immunofluorescent staining protocols [16–19] have questioned the model as well. In this study, we aimed to gain further insight into the interspecies conservation of 5mC and 5hmC patterns during the first cell cycle. To this end, we characterized for the first time the dynamics of 5mC and 5hmC throughout pronuclear development in equine zygotes produced by ICSI, independently in the maternal (mPN) and the paternal (pPN) pronucleus, using an immunofluorescent staining protocol optimized previously [20, 21].

## Results

### Zygote classification according to pronuclear morphology

In this study, the dynamic patterns of 5mC and 5hmC were analyzed independently for the pPN and the mPN during pronuclear development in in vitro-produced equine zygotes. To this end, the zygotes (*n* = 141) were classified into different stages according to size, position and conformation of the pronuclei. Due to the lipid-rich cytoplasm of equine oocytes and zygotes, immunostaining was used to identify the following pronuclear stages: (1) PN0: decondensing sperm head and meiosis II finished with the second polar body extruded and the chromosomes which start to decondense, (2) PN1: the decondensed DNA of the mPN and pPN is forming two small pronuclei, (3) PN2: the pronuclei are increasing in size and start to migrate toward the center, (4) PN3: the pronuclei reached their maximum size and are in apposition and (5) PN4: the pronuclei are in apposition and display an area with a fibrillary aspect.

**Fig. 1:**
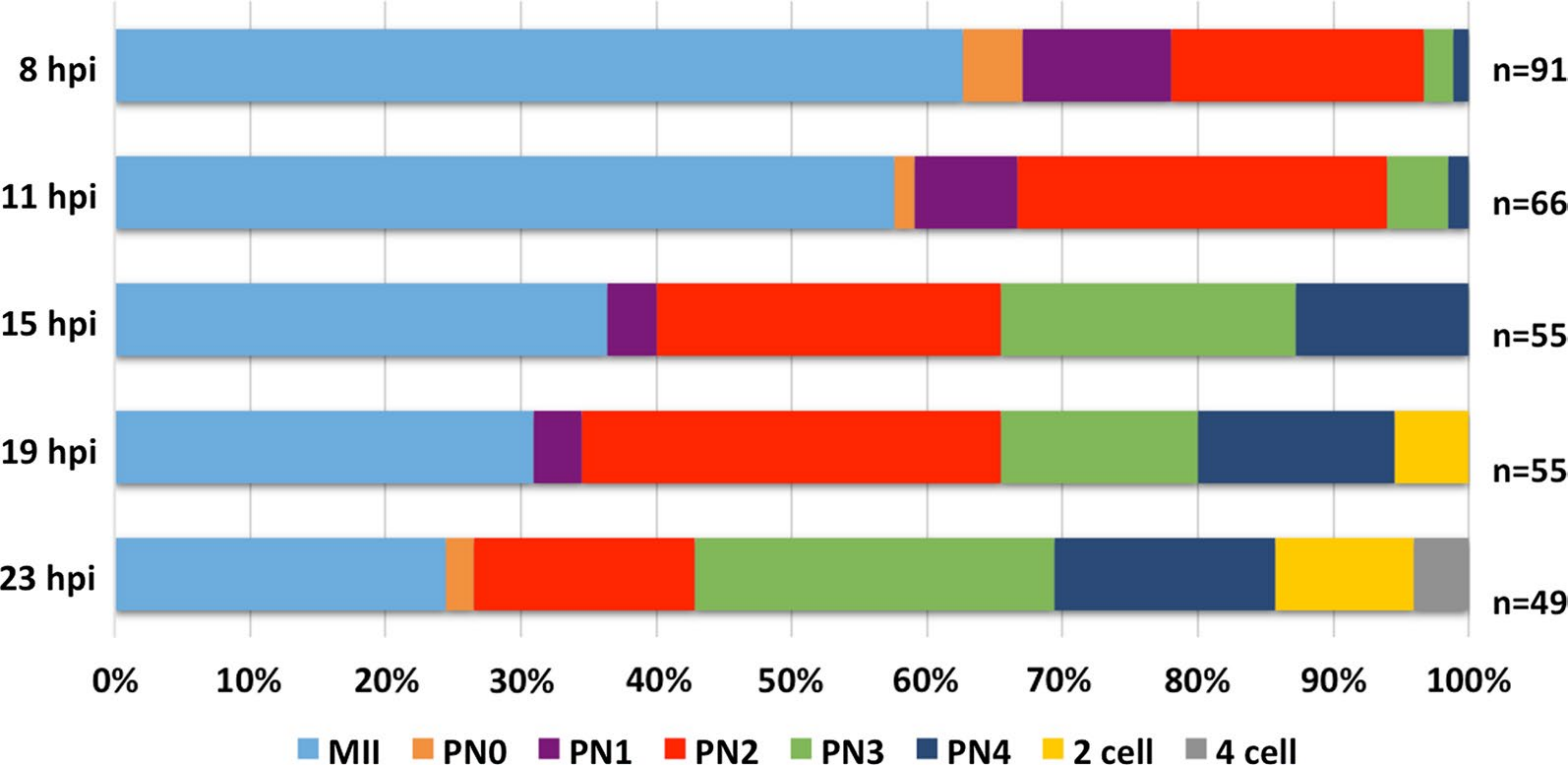
Distribution of ICSI-produced equine embryos among developmental stages in each time point of collection. The time points of collection are measured in hours after ICSI (hpi). The oocytes in metaphase II (MII) did not activate after the injection of the spermatozoon, which was found intact in the cytoplasm of the oocyte. The number of injected oocytes collected at each time point is indicated in the *right*. Degenerated oocytes and zygotes were excluded from the analysis

To obtain all pronuclear stages, equine zygotes were collected at five time points ranging from 8 to 23 h after fertilization using intracytoplasmic sperm injection (ICSI). The distribution of the pronuclear and cleavage stages among the different collection time points is illustrated in Fig. 1. As the oocytes were fertilized using ICSI, the moment of penetration of the spermatozoon was timed precisely. Nevertheless, a lot of variability in the developmental stage of the embryos within each collection time point was observed, indicating that the activation of the oocyte and further pronuclear formation in the individual embryos were not evolving synchronously.

The oocyte activation rate increased with time in culture (h). The shortest activation rate was observed at 8 h after ICSI, with less than 40% of the injected oocytes activated, while 75.5% was activated at 23 h after ICSI, i.e., the last time point of collection. At 8 h after ICSI, most zygotes were identified as PN1 (11%) and PN2 (18%) stages. At 11 h after ICSI, the majority of zygotes were at PN2 stage (27%), with only 8% and 3% at PN1 and PN3, respectively. At 15 h after ICSI, PN2 (26%) was again the most observed stage although 22% of the collected zygotes were in PN3. At 19 h after ICSI, zygotes were still mostly in PN2 (31%), while 15% of the zygotes were in either PN3 or PN4. At 23 h after ICSI, only 16% of the zygotes were in PN2 and PN3 became the most common stage (27%). Within these last two time points of collection, i.e., 19 and 23 h after ICSI, 5% and 10% of the embryos already reached the two-cell stage, respectively. Furthermore, 23 h after ICSI, 4% of the embryos were at the four-cell stage. PN1 might be considered as a very transitory stage as it was only observed in 19 of the 141 zygotes included in the study, while PN2, the stage in which the pronuclei grow and migrate, was present at all collection time points and can be considered as the longest stage with 59 of the 141 zygotes observed in that stage.

### Determination of the parental pronuclear origin and DNA content

**Fig. 2:**
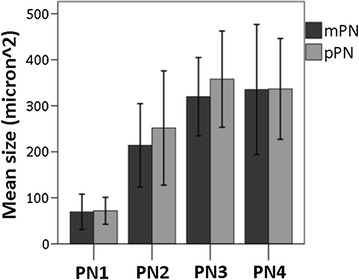
Evolution of the pronuclear size during development. No differences were found in mean pronuclear size (at the largest diameter) between the mPN and pPN in the four pronuclear stages. PN1 *n* = 19, PN2 *n* = 59, PN3 *n* = 37, PN4 *n* = 26. *Error bars* represent ±1 SD

In the mouse, the relative pronuclear size is used to determine the parental origin of the pronuclei [22, 23]. However, the relative size of the pronuclei in the horse is variable and does not differ between the mPN and pPN, as confirmed in this study (Fig. 2). Therefore, the asymmetric pattern of the tri-methylation of histone H3 lysine 9 (H3K9me3) was used to accurately differentiate between the mPN and pPN. This histone modification is in the horse only present in the mPN throughout pronuclear development, as described previously [20].

**Fig. 3:**
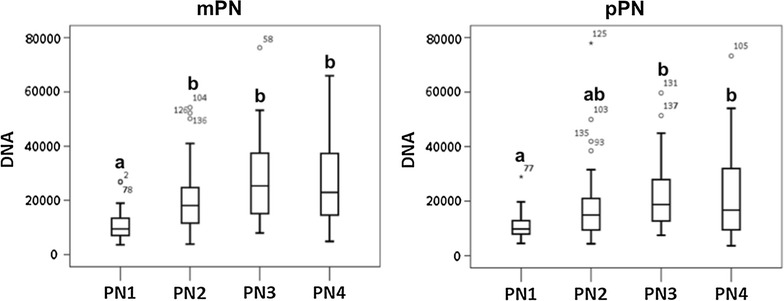
Total DNA fluorescence of the paternal (pPN) and maternal (mPN) pronucleus in each pronuclear stage. Comparisons were made between the different pronuclear stages independently for the pPN and the mPN; 141 zygotes were included in the study (PN1 *n* = 19, PN2 *n* = 59, PN3 *n* = 37 and PN4 *n* = 26). In the pPN, significant differences were observed between PN1 and PN3-PN4. In the mPN, significant differences were found between PN1 and PN2-PN3-PN4. The data were analyzed with SPSS Statistics 24, by the Kruskal–Wallis *H* test combined with Bonferroni correction for multiple testing. *Different superscripts* (*a* or *b*) indicate significant differences, and *p* values <0.05 were considered significant

After determination of the stage and parental origin of the pronuclei, the total DNA fluorescence was calculated for each pronucleus of each zygote by multiplying the fluorescence intensity by its corresponding pronuclear area. When the total DNA fluorescence between the different pronuclear stages was compared independently for the mPN and the pPN (Fig. 3), a significant increase was observed between PN1 and PN2 in the mPN (*p* value = 0.01) and between PN1 and PN3 in the pPN (*p* value = 0.01), which is indicative for DNA replication. In the rabbit, it has been demonstrated that DNA replication occurs during the migration and enlargement of the pronuclei (PN2 and PN3 of their classification) by injecting DIG-11dUTP in the zygotes [24]. Consequently, the total fluorescence of 5mC or 5hmC was divided by its corresponding total DNA fluorescence, as performed by Reis Silva et al. [24], to correct for the DNA replication that occurs during pronuclear development [25].

### Dynamics of DNA methylation during pronuclear development

The global DNA methylation pattern of 60 ICSI-produced equine zygotes was analyzed throughout pronuclear development (PN1: *n* = 6, PN2: *n* = 25, PN3: *n* = 18, PN4: *n* = 11).

**Fig. 4:**
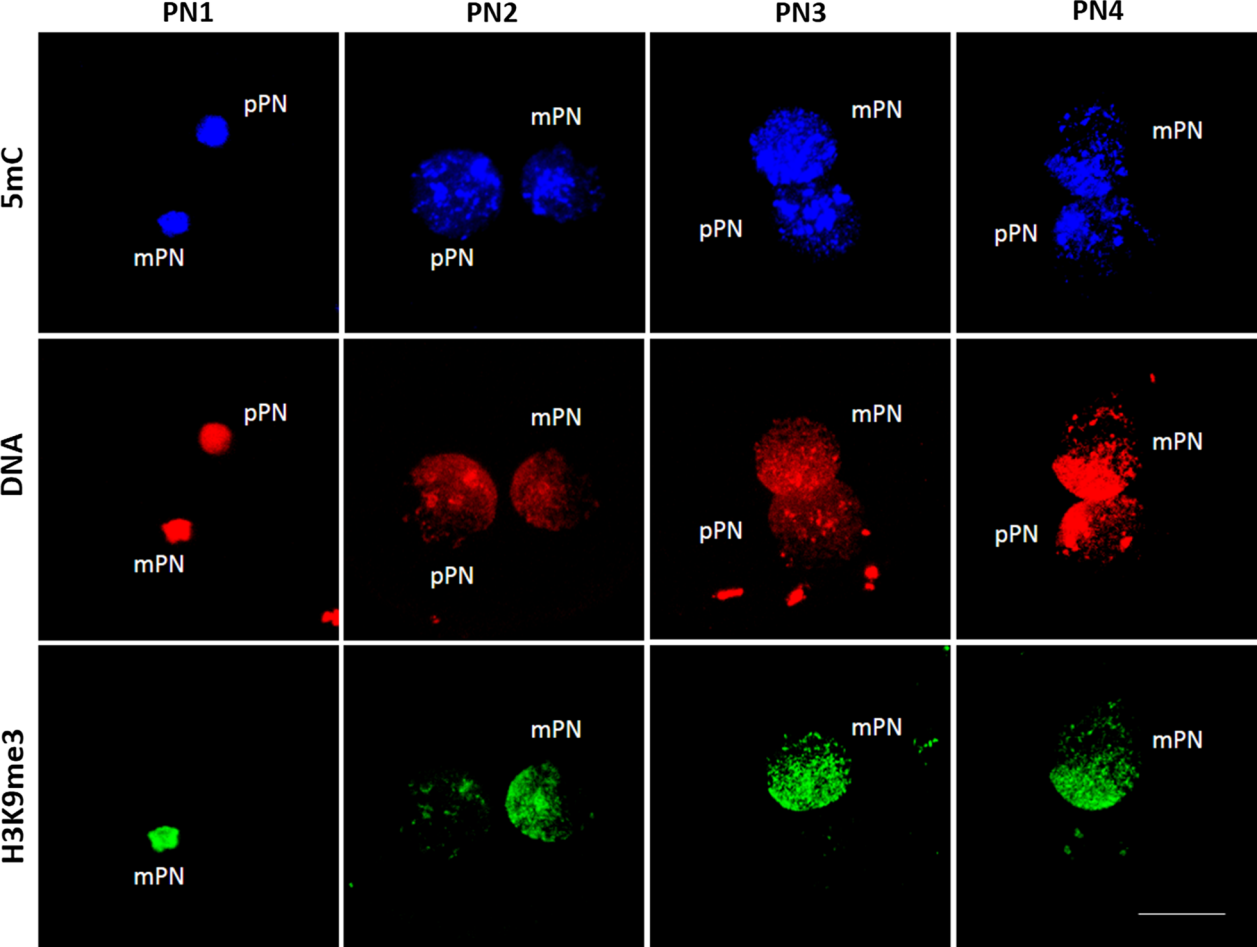
5-Methylcytosine (5mC) patterns in the maternal (mPN) and paternal (pPN) pronucleus during pronuclear development. 5mC, *in blue*, was clearly present throughout pronuclear development in both the mPN and the pPN. The mPN was identified by H3K9me3 immunostaining (*in green*). The DNA was stained by EthD-2 (*in red*). All the images were taken at 630× and the scale bar represents 20 μm

**Fig. 5:**
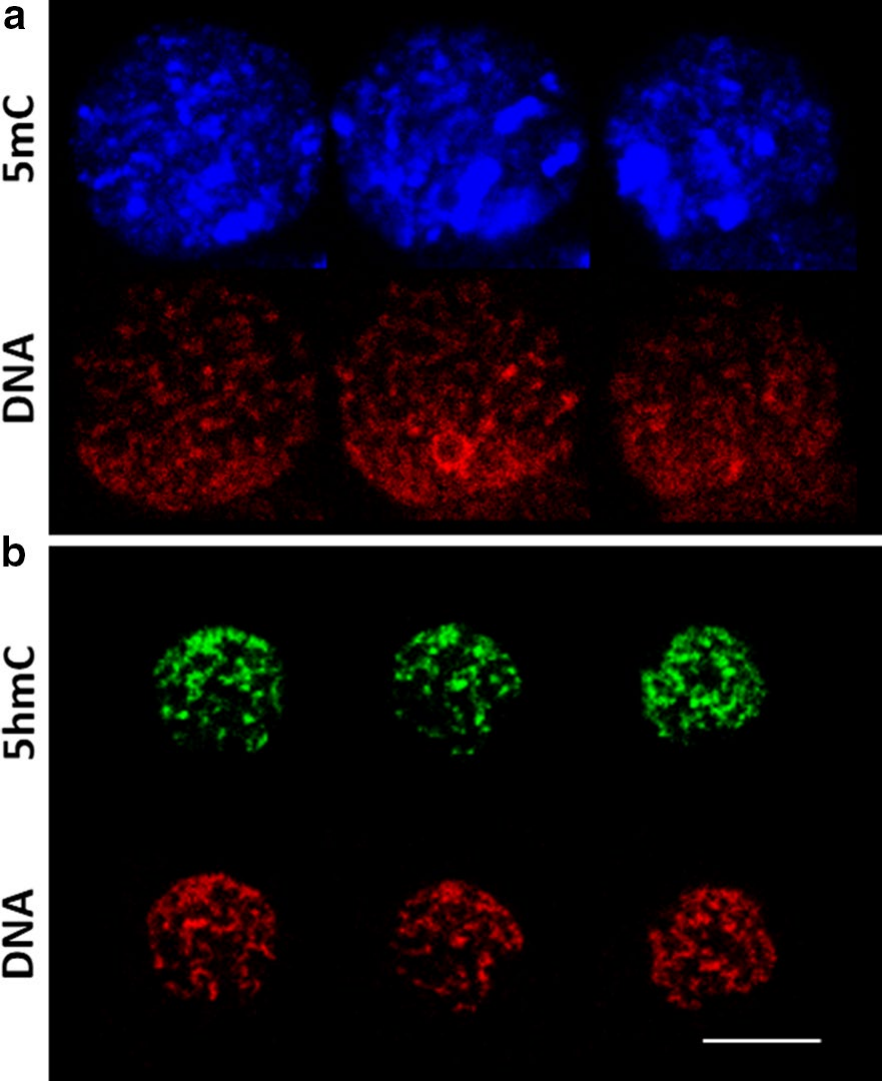
Distribution of 5-methylcytosine (5mC) and 5-hydroxymethylcytosine (5hmC) within the pronucleus. Single images were obtained at different positions of the same pronucleus from z-stacks to illustrate the distribution of 5mC and 5hmC. The pattern of distribution of 5mC and 5hmC differs largely, with 5hmC being distributed homogeneously, while 5mC was concentrated in specific DNA regions. 5mC and 5hmC were evaluated in different zygotes

5mC was highly present in both the mPN and the pPN and no differences in intensity could be determined at a glance between the mPN and the pPN (Fig. 4). Interestingly, the distribution of 5mC in the pronuclei was rather heterogeneous with higher concentrations in certain regions of the DNA (Fig. 5a).

**Fig. 6:**
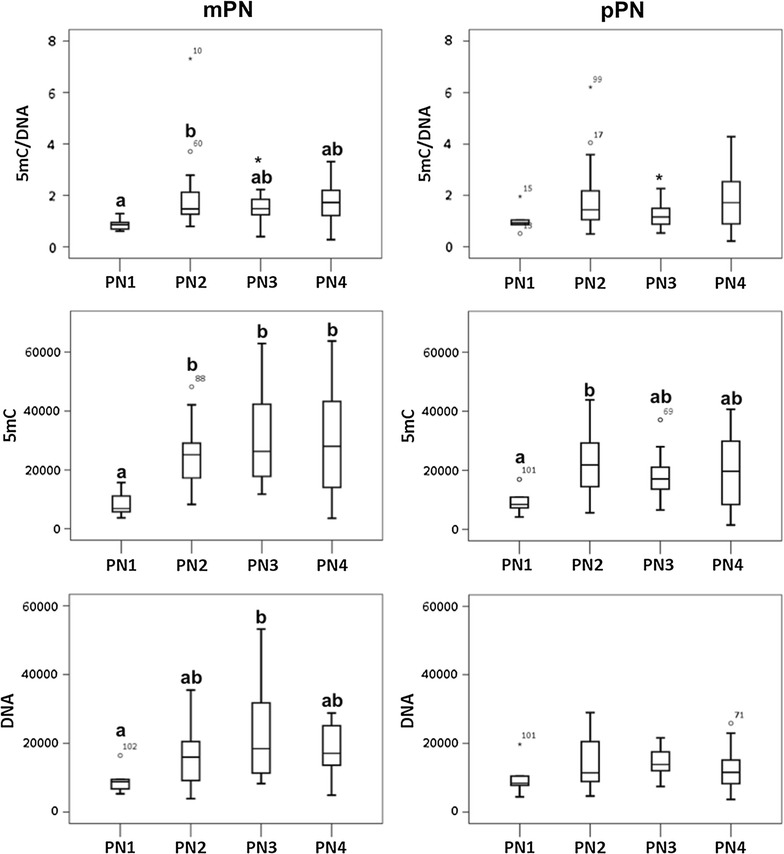
Dynamics of global DNA methylation in the paternal (pPN) and the maternal (mPN) pronucleus. In the pPN, no differences were found either in the normalized 5mC (5mC/DNA) or in the total DNA fluorescence (DNA) between pronuclear stages. However, an increase in the total 5mC fluorescence (5mC) was observed between PN1 and PN2. In the mPN, an increase in the normalized 5mC (5mC/DNA) was found between PN1 and PN2. Similarly, an increase in the total 5mC fluorescence (5mC) was found between PN1 and PN2–PN3–PN4, and in the total DNA fluorescence (DNA) between PN1 and PN3. Finally, higher levels of normalized 5mC (5mC/DNA) were found in the mPN than the pPN at PN3 (*). The distribution of the 60 embryos included in the study was: PN1 (*n* = 6), PN2 (*n* = 25), PN3 (*n* = 18) and PN4 (*n* = 11). *Different superscripts* (*a* or *b*) indicate significant differences, and *p* values <0.05 were considered significant

The dynamics of the normalized 5mC fluorescence (5mC/DNA) between the different pronuclear stages showed a significant increase between PN1 and PN2 in the mPN (*p* value = 0.022) (Fig. 6). In the pPN, on the other hand, no significant differences in normalized 5mC fluorescence were observed between pronuclear stages. Regarding total 5mC fluorescence, a significant increase was found between PN1 and PN2, PN3, and PN4 in the mPN (*p* value = 0.015, 0.003 and 0.011, respectively) and between PN1 and PN2 in the pPN (*p* value = 0.032). The total DNA fluorescence increased significantly between PN1 and PN3 (*p* value = 0.023) in the mPN, but the increase in DNA fluorescence observed in the pPN when all zygotes included in the study were taken into account (5mC + 5hmC: *n* = 141) (Fig. 3) did not reach statistical significance when only the 5mC-stained pPN (*n* = 60) was considered (Fig. 6). Finally, higher normalized 5mC fluorescence was observed in the mPN than in the pPN at PN3, with a paternal/maternal ratio of 0.83 (*p* value = 0.046).

### Dynamics of DNA hydroxymethylation during pronuclear development

To evaluate the global hydroxymethylation pattern in equine zygotes, 81 zygotes were produced using ICSI and distributed in four pronuclear stages (PN1: *n* = 13, PN2: *n* = 34, PN3: *n* = 19, PN4: *n* = 15).

**Fig. 7:**
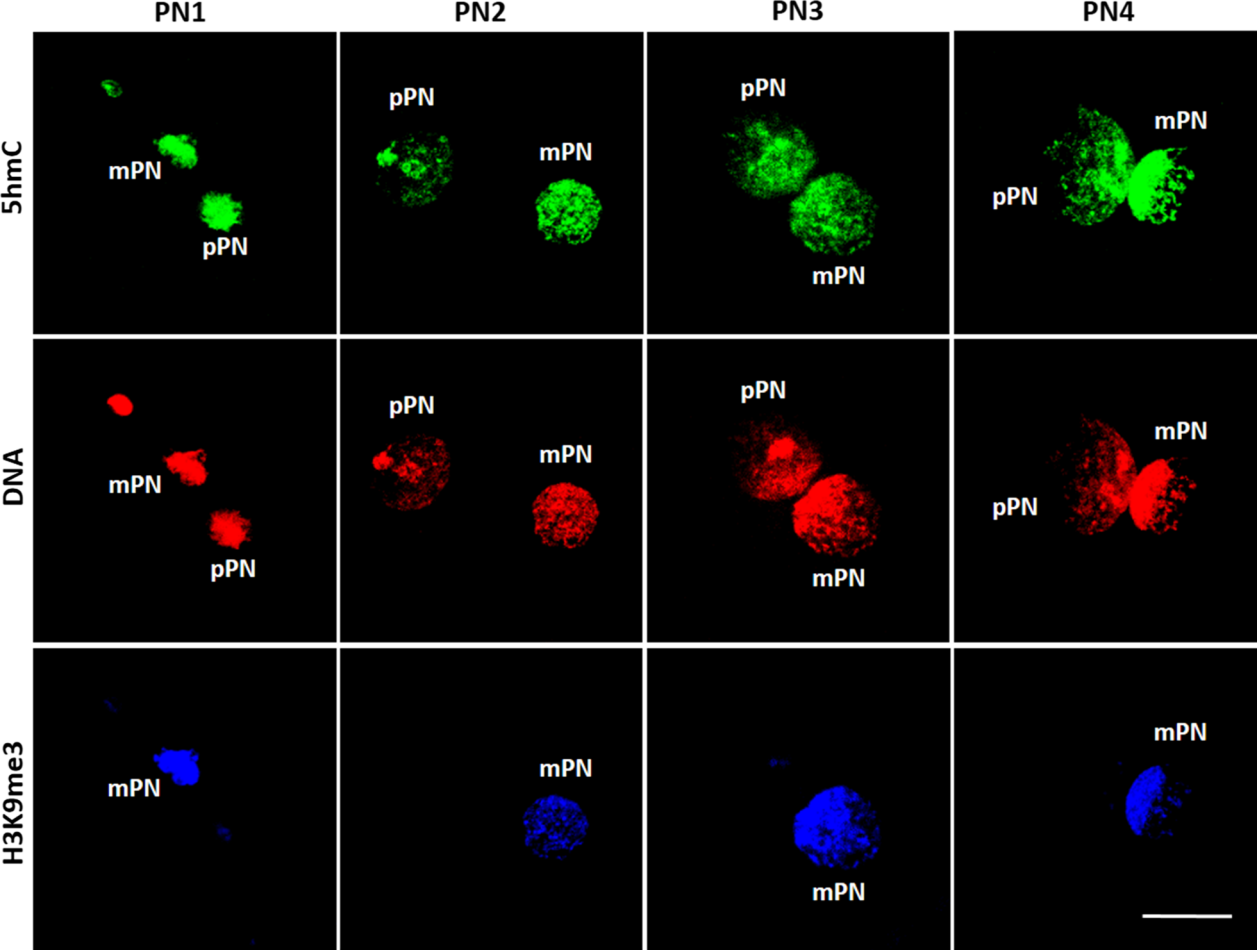
5-Hydroxymethylcytosine (5hmC) patterns in the maternal (mPN) and paternal (pPN) pronucleus during pronuclear development. 5hmC, *in green*, was clearly present throughout pronuclear development in both the mPN and the pPN. The parental origin of the pronuclei was determined by H3K9me3 immunostaining (*in blue*) and the DNA was stained by EthD-2 (*in red*). All the images were taken at 630×. The *scale bar* represents 20 μm

**Fig. 8:**
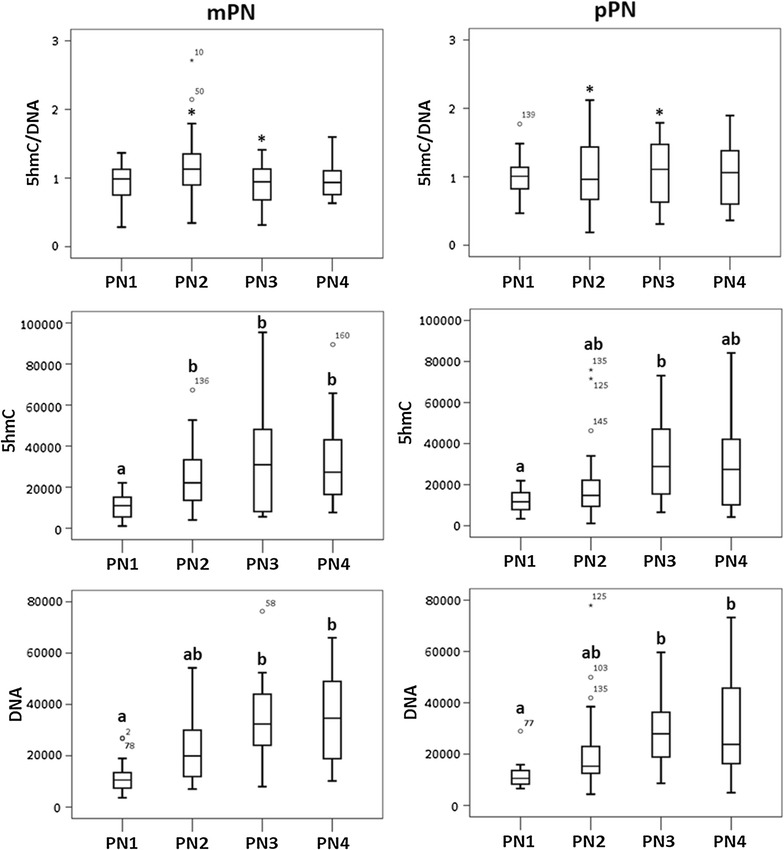
Dynamics of global DNA hydroxymethylation in the paternal (pPN) and the maternal (mPN) pronucleus. No differences in the normalized levels of 5hmC (5hmC/DNA) were observed between the pronuclear stages in pPN or the mPN. However, the levels of normalized 5hmC were significantly higher in the maternal PN2 than its paternal counterpart and in the paternal PN3 than its maternal counterpart (*). A significant increase in the levels of total 5hmC (5hmC) was found in the pPN between PN1 versus PN3 and in the mPN between PN1 vs. PN2–PN3–PN4. Additionally, a significant increase in the total DNA fluorescence (DNA) was observed between PN1 and PN3–PN4, in both pPN and mPN. The distribution of the 81 embryos included in the study was: PN1 (*n* = 13), PN2 (*n* = 34), PN3 (*n* = 19) and PN4 (*n* = 15). *Different superscripts* (*a* or *b*) indicate significant differences, and *p* values <0.05 were considered significant

In all the zygotes analyzed, 5hmC was highly present throughout pronuclear development in both the pPN and mPN, with no observable differences depending on the parental pronuclear origin (Fig. 7). In contrast to the distribution of 5mC, 5hmC was very homogeneously distributed in the DNA (Fig. 5b). After quantifying the fluorescence intensity, no significant differences were observed in the normalized 5hmC levels (5hmC/DNA) between pronuclear stages regardless the parental origin of the pronuclei (Fig. 8), indicating that the ratio of 5hmC to DNA is relatively constant throughout pronuclear development. The total 5hmC fluorescence showed a significant increase between PN1 and PN2, PN3 and PN4 (*p* value = 0.025, 0.009 and 0.004, respectively) in the mPN, and between PN1 and PN3 (*p* value = 0.008) in the pPN (Fig. 8). Analogously, the total DNA fluorescence between pronuclear stages also increased significantly between PN1 and PN3-PN4 in both the mPN (*p* value = 0.001) and the pPN (*p* value = 0.01 and 0.002, respectively). These results indicate that the increase in 5hmC is associated with the increase in DNA. Significantly lower levels of normalized 5hmC were found in the pPN compared to the mPN in PN2, with a paternal/maternal ratio of 0.87 (*p* value = 0.027). The opposite situation was observed in PN3, with significantly higher levels of normalized 5hmC in the pPN compared to the mPN and a paternal/maternal ratio of 1.25 (*p* value = 0.027).

## Discussion

In this study, we analyzed for the first time the global dynamic patterns of 5mC and 5hmC independently for the pPN and the mPN during pronuclear development in equine zygotes produced by ICSI.

To evaluate the dynamic changes in 5mC and 5hmC during pronuclear development, equine zygotes were collected at five different time points within the first 23 h after ICSI. Within this time frame, we were able to assess the whole pronuclear development as the embryo stages collected ranged from injected oocytes, which were not activated yet to four-cell embryos.

The presence of 5mC and 5hmC was evaluated by immunofluorescent staining in individual embryos. Although this is a very powerful tool to study the global changes in 5mC and 5hmC levels, the results are highly influenced by the protocol used, with the proper exposure of the epitopes and the optimal incubation time and concentration of the primary antibodies being of major importance [18]. For example, it has been demonstrated in mouse zygotes that a reduction in the concentration of 5hmC primary antibody resulted in a reduced signal in the mPN [18], and the incubation with 5hmC primary antibody for longer than 1 h at room temperature or shorter than 6 h at 4 °C resulted in higher levels in the pPN than in the mPN, while these differences were no longer observed when saturation binding conditions were used [17]. Additionally, when epitopes are retrieved using the traditional acid-based method, an active demethylation in mouse zygotes is observed resulting in complete DNA demethylation of the pPN as early as 8 h after fertilization [4, 11, 15, 23, 26]. However, if a new method to retrieve the 5mC epitope in mice is used, which combines the traditional acid treatment with a short tryptic digestion, high levels of 5mC in both pronuclei throughout pronuclear development are observed instead of the active demethylation of the pPN [22].

To overcome this bias, we optimized the immunofluorescent staining in equine zygotes in a previous study, in which the traditional acid treatment (4 N HCl) was compared to the new epitope retrieval method (4 N HCl + tryptic digestion) to evaluate the dynamics of 5mC and 5hmC and no significant differences between both methods were observed [27]. The new epitope retrieval method was chosen in this case because it reduces the acid treatment time by half. Furthermore, the optimal concentration and incubation time for the primary antibodies were determined.

The different accessibility of 5mC and 5hmC antibodies to the DNA depending on its parental origin in the mouse, evidenced by the asymmetric pattern of this marks observed between the mPN and pPN after short incubation with the primary antibodies or different epitope retrieval methods, indicates a different chromatin conformation depending on its parental origin. Indeed, the paternal DNA undergoes massive changes during pronuclear development, which might explain the different conformation. In the horse, however, this possible asymmetric chromatin conformation does not affect the binding of 5mC and 5hmC antibodies.

During the first cell cycle, DNA replication takes place [25] and the size and complexity of the pronuclei vary enormously, which renders fluorescence intensity quantification even more challenging. In order to minimize these influences, we carefully classified the collected zygotes into four different pronuclear stages, based on the size and the conformation of their pronuclei. Additionally, the normalized fluorescence of 5mC or 5hmC was calculated for each pronucleus to correct for DNA replication.

In this study, we found no evidence of active DNA demethylation of the pPN in the horse. 5mC was highly present in both the mPN and pPN throughout pronuclear development. In the mPN, we found only increased normalized 5mC levels between PN1 and PN2, but no differences were further found with PN3 and PN4, reflecting stable normalized 5mC levels from PN1 to PN4. These observations are in contrast to the classic model of global methylation erasure of the pPN during the first cell cycle as established in mouse [4] and conserved in human [7, 11] and rat [6, 28]. However, they are in line with the results obtained in other species including rabbits [8, 24], sheep [11], pigs [9] and goats [10], where no loss of methylation was reported neither in the pPN or the mPN during the first cell cycle.

At first, 5hmC was considered as being only an intermediate form for DNA demethylation through TET oxidation [12]. This hypothesis was supported by the observed inverse patterns between 5mC and 5hmC reported in mouse, rabbit and bovine zygotes, where increasing 5hmC levels in the pPN was accompanied by decreasing 5mC levels [5, 15, 29]. Nevertheless, it must be mentioned that some studies in mouse, using different immunostaining protocols, were not able to confirm this inverse pattern [16, 17]. Also in this study, this inverse relationship between 5mC and 5hmC was not observed, and 5mC as well as 5hmC coexisted in both the mPN and pPN throughout pronuclear development in the horse. Even though 5mC and 5hmC levels were not studied within the same embryo, and consequently, we were not able to correlate the dynamics of both marks, we did observe a completely different distribution pattern. While 5mC seemed more concentrated in some DNA regions, 5hmC was homogeneously distributed in the pronuclear DNA. This different distribution pattern of 5mC and 5hmC has also been observed in cleavage stage embryos in the mouse, especially in the emerging pluripotent lineage where the presence of 5mC was restricted to some intensely stained foci [30]. This asymmetric pattern might be a consequence of the strong association of 5mC to heterochromatin, while 5hmC is preferentially associated with euchromatin [30]. Moreover, this finding also supports the growing evidence that 5hmC plays its own epigenetic role [16, 17, 31, 32].

It has been previously reported in mice, rabbit and cattle [5, 15, 16] that 5hmC is only present (or present in a consistent higher level) in the pPN compared to its maternal counterpart. This was not observed in the horse, where high levels of 5hmC were found in both mPN and pPN. In this study, higher levels of 5hmC were demonstrated in the maternal PN2 compared to the paternal counterpart and vice versa in PN3: This is probably due to an intrinsic artifact of the fluorescence intensity quantification rather than to a biological explanation, especially when considering the high complexity of the pronuclei. Additionally, no differences were observed in normalized 5hmC levels between pronuclear stages regardless of the parental origin of the pronuclei in the horse, evidencing a stable presence of 5hmC during pronuclear development. This persistent level of 5hmC throughout pronuclear development in both pPN and mPN has also been reported in mouse [17] and human [19].

In cattle, a strong association between the pattern of H3K9me3 and of 5mC in the pPN was reported, where during pronuclear development, the presence of both marks simultaneously increased in the pPN [33]. It was hypothesized that de novo H3K9 methylation directed the de novo DNA methylation, and as such, in cattle, global demethylation of the pPN is followed by a gradual H3K9 methylation and finally DNA methylation [33]. In the horse, this association was not observed. Instead, 5mC levels remained high and constant during pronuclear development in the pPN, with no evidence of demethylation, while H3K9me3 was never present in the pPN, as previously demonstrated in the horse [20] and rabbit zygotes [24].

In conclusion, there is no evidence of global demethylation, nor active nor passive, in the horse during the first cell cycle. The normalized 5mC levels were high and stable during pronuclear development, showing no evidence of passive demethylation associated with DNA replication in the PN regardless their parental origin. Moreover, no evidence of active demethylation (replication independent and TET mediated) was observed as stable and non-complementary patterns of normalized 5mC and 5hmC were present in both PN during pronuclear development. However, it is important to keep in mind that the embryos used in this study were produced in vitro by ICSI. In some species, such as rabbit [24] and rat [28], the loss of methylation observed by immunostaining in the pPN of in vivo-derived zygotes, was reduced after in vitro culture [24], IVF [28] and more dramatically after ICSI [28]. In sheep [11] and cattle [34], on the other hand, no differences in DNA methylation patterns were observed between in vivo and IVF [11, 34] or ICSI [34] zygotes using the same immunostaining technique. In mouse, reduced loss of methylation of the pPN was observed after IVF when the acid-based epitope retrieval was used for immunostaining. When the acid-tryptic-based epitope retrieval was used instead, no differences in DNA methylation pattern were observed between in vivo-derived and in vitro-produced mouse zygotes, with both mPN and pPN showing high levels of DNA methylation [22]. Whether the results of this study apply for equine in vivo zygotes as well remains to be investigated. The collection of equine zygotes in vivo is challenging, since the horse is a monovulatory species that not responds to superovulation and as such, only one zygote at a time could be on average surgically collected. On the other hand, the ICSI protocol used in this study yields a 20% blastocyst rate, which is in line with the results obtained by other groups [35]. Additionally, healthy foals have been born from embryos produced using this protocol. Consequently, an active demethylation of the pPN during first cell cycle might not be essential to obtain normal, healthy offspring in the horse. In line with this observation, the use of round spermatid injection (ROSI) in mice leads to similar high levels of 5mC in the pPN and the mPN, with the zygotes being still able to develop to term [36]. This indicates that active demethylation of the pPN is not essential for normal embryo development in the mouse as well.

## Conclusions

In the present study, we described, for the first time in the horse, the global dynamics of DNA methylation and hydroxymethylation throughout pronuclear development, independently in the paternal and maternal genome.

No evidence of active demethylation of the paternal genome was found in the horse. Instead, 5mC and 5hmC coexisted at high levels in both parental genomes throughout pronuclear development. Moreover, 5mC and 5hmC displayed different distribution patterns in the pronuclei with 5hmC homogeneously distributed, while 5mC was more clustered within certain DNA regions. This suggests that both marks provide their own epigenetic information. Considering these results, global DNA demethylation during the first cell division might not be essential for embryo development in the horse.

## Methods

### Equine in vitro embryo production

Equine zygotes were produced in vitro by piezo drill-assisted ICSI as described previously [37]. Briefly, ovaries were collected from slaughtered mares and processed within 4 h. Cumulus oocyte complexes (COCs) were aspirated from follicles larger than 5 mm using a 16-gauge needle attached to a vacuum pump (−100 mm Hg) and matured in groups of maximum 30 in 500 µL of Dulbecco’s modified Eagle medium nutrient mixture F-12 (DMEM/F12; Gibco)-based maturation medium [38] for minimum 25 h at 38.5 °C in a humidified atmosphere of 5% CO_2_ in air. After maturation, COCs were denuded by gentle pipetting in 0.05% bovine hyaluronidase (Sigma-Aldrich) for two minutes, followed by further pipetting in HEPES-buffered TCM199 medium (Gibco) supplemented with 10% fetal bovine serum (FBS). Only oocytes with an extruded polar body were used for piezo drill-assisted ICSI. Frozen and fresh sperm of two different stallions was used for ICSI; after Percoll gradient centrifugation, the sperm was washed and kept in calcium-free TALP and manipulated in 9% polyvinylpyrrolidone (Sigma-Aldrich) in phosphate-buffered saline (PBS, Gibco). All manipulations were performed on the heated stage (37 °C) of an inverted microscope; a progressively motile sperm was immobilized by piezo pulses and subsequently injected into the cytoplasm of a mature oocyte using a piezo drill. The injected oocytes were cultured in groups of 10–15 in 20 μl drops of DMEM-F12 supplemented with 10% FBS (Greiner Bio-One) and 50 µg/ml Gentamycin (Sigma) at 38.2 °C in a humidified atmosphere of 5% CO_2_, 5% O_2_ and 90% N_2_. Presumptive zygotes were collected after 8, 11, 15, 19 and 23 h of culture in order to obtain all the pronuclear stages.

### Immunofluorescent staining

After collection, presumptive zygotes were vortexed for 1 min to remove any remaining cumulus cells, fixed in 4% paraformaldehyde (PFA; Sigma-Aldrich) for 20 min at room temperature and kept in 2% PFA at 4 °C for a maximum of four days until immunostaining was performed. The zygotes were subsequently washed in PBS containing 0.5% bovine serum albumin (BSA, Sigma-Aldrich) for 1 h at room temperature. After washing, they were permeabilized with 0.5% Triton X-100 (Sigma-Aldrich) and 0.05% Tween 20 (Sigma-Aldrich) in PBS for 1 h at room temperature, and washed three times for 5 min in 0.5% BSA in PBS. Zygotes were blocked in PBS containing 2% BSA for 1 h at room temperature and washed three times for 2 min in 0.5% BSA in PBS. After washing, they were incubated with the primary antibody rabbit anti-H3K9me3 (1:100, Active Motif) in 2% BSA in PBS overnight at 4 °C. Simultaneously, four zygotes were incubated with the non-immune control antibody rabbit IgG (0.01 mg/ml; Rockland 011-0102) in 2% BSA in PBS overnight at 4 °C. Zygotes which served as negative control remained in 2% PBS in BSA without adding any primary antibody. Next, all zygotes, including the non-immune and the negative control, were washed three times for 10 min in 0.5% BSA in PBS, post-fixed in 4% PFA for 25 min and washed again three times in 0.5% BSA in PBS for 10 min. Epitope retrieval was performed as described previously [20] by treating the zygotes with 4 N HCL (Sigma-Aldrich) for 30 min at room temperature and with 100 mM Tris–HCl (pH 8.5; Sigma-Aldrich) for 10 min at room temperature, followed by three times washing with 5% BSA in PBS for 5-min and 20-s treatment with 0.25% (w/v) trypsin (Sigma-Aldrich T4799) at 37 °C. Tryptic digestion was stopped by incubating the zygotes with 30% goat serum (Gibco) in PBS for 2 min at room temperature and subsequently washing them three times with 0.5% BSA in PBS for 5 min. After epitope retrieval, zygotes were treated with 1 mg/mL RNase A (Affymetrix) for 30 min at 37 °C to avoid the binding of the nuclear stain, ethidium homodimer 2 (EthD-2; Molecular Probes), to RNA [39]. Subsequently, they were washed three times in 0.5% BSA in PBS for 5 min and incubated with the nuclear stain 0.5 nM EthD-2 in 0.5% BSA in PBS for 30 min at room temperature. After four washing steps of 2 min in 0.5% BSA in PBS, zygotes were incubated in blocking solution overnight at 4 °C. Subsequently, zygotes previously incubated with rabbit anti-H3K9me3 were incubated with either mouse anti-5mC (0.01 mg/ml; EpiGentek A-1014-050) or mouse anti-5hmC (0.01 mg/ml; Active Motif 39999) primary antibodies in 30% goat serum in PBS overnight at 4 °C. At the same time, zygotes previously incubated with rabbit IgG control antibody were incubated with mouse IgG control antibody (0.01 mg/ml; Sigma-Aldrich) in 30% goat serum in PBS overnight at 4 °C. Zygotes used as negative control were placed in 30% goat serum in PBS without the addition of primary antibodies. After incubation with the primary antibodies, zygotes were washed and serially incubated with the two secondary antibodies in 30% goat serum in PBS, for 1 h at room temperature each. For the H3K9me3-5mC immunostaining, goat anti-rabbit FITC (0.02 mg/ml; Life Technologies) and goat anti-mouse Alexa Fluor 405 (0.02 mg/ml; Abcam) secondary antibodies were used, respectively. For the H3K9me3-5hmC immunostaining, goat anti-rabbit Alexa Fluor 405 (0.02 mg/ml; Abcam) and goat anti-mouse Alexa Fluor 488 (0.02 mg/ml; Abcam) secondary antibodies were used, respectively. After four washes of 5 min in 0.5% BSA in PBS, two zygotes were mounted per slice in 24.7 mg/ml 1,4-diazabicyclo[2.2.2]octane (DABCO; Sigma-Aldrich) in 90% glycerol and 10% PBS, to prolong the lifetime of the dyes.

### Fluorescence microscopy and image analysis

To avoid fading, evaluation of the embryos was performed 2 days after immunostaining was completed, using a Leica TSC SPE-II confocal microscope (Leica, Belgium) with an ACS APO 63× oil immersion objective (Leica) and laser lines at 405, 488 and 561 nm wavelengths.

For each wavelength, single images were taken independently of the equatorial cross section of each pronucleus. Additionally, digital optical sections of the area containing both pronuclei were taken for each wavelength using Z-series acquisition feature every 0.5 µm.

Quantitative analysis of fluorescence intensities was performed using the ImageJ software.

The area of the equatorial cross section of each pronucleus was manually outlined and measured in the single images. After subtracting the background, the mean fluorescence intensity was measured for 5mC, 5hmC and EthD-2. The mean fluorescence intensities were then multiplied by the pronuclear areas to obtain the total fluorescence of 5mC, 5hmC and EthD-2. Finally, for each pronucleus, the total fluorescence of 5mC or 5hmC was divided by the total fluorescence of EthD-2 to obtain the normalized fluorescence.

When a small part of the mPN and pPN was overlapping, the overlapping area was not measured. Additionally, when most of the area of the pronuclei was overlapping and the analysis of both pronuclei could not be made independently, the zygotes were excluded from the study.

### Statistical analysis

Four replicates were performed for 5mC and 5hmC. The Kruskal–Wallis *H* test combined with Bonferroni correction for multiple testing was used to compare the total and normalized fluorescence between the different pronuclear stages, independently for the mPN and pPN. The paired samples *t* test was used to compare the size and the normalized fluorescence between the mPN and the pPN in each pronuclear stage. All the analyses were performed with SPSS Statistics 24 and *p* values <0.05 were considered significant.

## Abbreviations

TET: ten-eleven translocation enzymes
5mC: 5-methylcytosine
5hmC: 5-hydroxymethylcytosine
5fC: 5-formylcytosine
5caC: 5-carboxylcytosine
H3K9me2: histone H3 lysine 9 di-methylation
ICSI: intracytoplasmic sperm injection
mPN: maternal pronucleus
pPN: paternal pronucleus
H3K9me3: histone H3 lysine 9 tri-methylation
ROSI: round spermatid injection
COCs: cumulus oocytes complexes
DMEM/F12: Dulbecco’s modified Eagle medium nutrient mixture F-12
FBS: fetal bovine serum
PBS: phosphate-buffered saline
PFA: paraformaldehyde
BSA: bovine serum albumin
EthD-2: ethidium homodimer 2
DABCO: 1,4-diazabicyclo[2.2.2]octane

## References

[1] Reik W, Dean W, Walter J (2001). Epigenetic reprogramming in mammalian development. Science.

[2] Dean W, Santos F, Stojkovic M, Zakhartchenko V, Walter J, Wolf E (2001). Conservation of methylation reprogramming in mammalian development: aberrant reprogramming in cloned embryos. Proc Natl Acad Sci USA.

[3] Oswald J, Engemann S, Lane N, Mayer W, Olek A, Fundele R (2000). Active demethylation of the paternal genome in the mouse zygote. Curr Biol.

[4] Mayer W, Niveleau A, Walter J, Fundele R, Haaf T (2000). Demethylation of the zygotic paternal genome. Nature.

[5] Iqbal K, Jin SG, Pfeifer GP, Szabo PE (2011). Reprogramming of the paternal genome upon fertilization involves genome-wide oxidation of 5-methylcytosine. Proc Natl Acad Sci USA.

[6] Zaitseva I, Zaitsev S, Alenina N, Bader M, Krivokharchenko A (2007). Dynamics of DNA-demethylation in early mouse and rat embryos developed in vivo and in vitro. Mol Reprod Dev.

[7] Xu Y, Zhang JJ, Grifo JA, Krey LC (2005). DNA methylation patterns in human tripronucleate zygotes. Mol Hum Reprod.

[8] Shi W, Dirim F, Wolf E, Zakhartchenko V, Haaf T (2004). Methylation reprogramming and chromosomal aneuploidy in in vivo fertilized and cloned rabbit preimplantation embryos. Biol Reprod.

[9] Jeong YS, Yeo S, Park JS, Koo DB, Chang WK, Lee KK (2007). DNA methylation state is preserved in the sperm-derived pronucleus of the pig zygote. Int J Dev Biol.

[10] Hou J, Lei TH, Liu L, Cui XH, An XR, Chen YF (2005). DNA methylation patterns in in vitro-fertilised goat zygotes. Reprod Fertil Dev.

[11] Beaujean N, Hartshorne G, Cavilla J, Taylor J, Gardner J, Wilmut I (2004). Non-conservation of mammalian preimplantation methylation dynamics. Curr Biol.

[12] Tahiliani M, Koh KP, Shen Y, Pastor WA, Bandukwala H, Brudno Y (2009). Conversion of 5-methylcytosine to 5-hydroxymethylcytosine in mammalian DNA by MLL partner TET1. Science.

[13] Kriaucionis S, Heintz N (2009). The nuclear DNA base 5-hydroxymethylcytosine is present in Purkinje neurons and the brain. Science.

[14] Szabo PE, Pfeifer GP (2012). H3K9me2 attracts PGC7 in the zygote to prevent Tet3-mediated oxidation of 5-methylcytosine. J Mol Cell Biol.

[15] Wossidlo M, Nakamura T, Lepikhov K, Marques CJ, Zakhartchenko V, Boiani M (2011). 5-Hydroxymethylcytosine in the mammalian zygote is linked with epigenetic reprogramming. Nat Commun.

[16] Salvaing J, Aguirre-Lavin T, Boulesteix C, Lehmann G, Debey P, Beaujean N (2012). 5-Methylcytosine and 5-hydroxymethylcytosine spatiotemporal profiles in the mouse zygote. PLoS ONE.

[17] Li Y, O’Neill C (2013). 5′-Methylcytosine and 5′-hydroxymethylcytosine each provide epigenetic information to the mouse zygote. PLoS ONE.

[18] Salvaing J, Li Y, Beaujean N, O’Neill C (2014). Determinants of valid measurements of global changes in 5′-methylcytosine and 5′-hydroxymethylcytosine by immunolocalisation in the early embryo. Reprod Fertil Dev.

[19] Petrussa L, Van de Velde H, De Rycke M (2016). Similar kinetics for 5-methylcytosine and 5-hydroxymethylcytosine during human preimplantation development in vitro. Mol Reprod Dev.

[20] Heras S, Smits K, Leemans B, Van Soom A (2015). Asymmetric histone 3 methylation pattern between paternal and maternal pronuclei in equine zygotes. Anal Biochem.

[21] Heras S, Forier K, Rombouts K, Braeckmans K, Van Soom A (2014). DNA counterstaining for methylation and hydroxymethylation immunostaining in bovine zygotes. Anal Biochem.

[22] Li Y, O’Neill C (2012). Persistence of cytosine methylation of DNA following fertilisation in the mouse. PLoS ONE.

[23] Santos F, Hendrich B, Reik W, Dean W (2002). Dynamic reprogramming of DNA methylation in the early mouse embryo. Dev Biol.

[24] Reis Silva AR, Adenot P, Daniel N, Archilla C, Peynot N, Lucci CM (2011). Dynamics of DNA methylation levels in maternal and paternal rabbit genomes after fertilization. Epigenetics.

[25] Hyttel P, Greve T, Callesen H (1989). Ultrastructural aspects of oocyte maturation and fertilization in cattle. J Reprod Fertil Suppl.

[26] Barton SC, Arney KL, Shi W, Niveleau A, Fundele R, Surani MA (2001). Genome-wide methylation patterns in normal and uniparental early mouse embryos. Hum Mol Genet.

[27] Heras S, Smits K, Van Soom A. Optimization of DNA methylation immunostaining in equine zygotes produced after ICSI. *EpiConcept COST Workshop* 2014:1.

[28] Yoshizawa Y, Kato M, Hirabayashi M, Hochi S (2010). Impaired active demethylation of the paternal genome in pronuclear-stage rat zygotes produced by in vitro fertilization or intracytoplasmic sperm injection. Mol Reprod Dev.

[29] Zhang P, Su L, Wang Z, Zhang S, Guan J, Chen Y (2012). The involvement of 5-hydroxymethylcytosine in active DNA demethylation in mice. Biol Reprod.

[30] Li Y, Seah MK, O’Neill C (2016). Mapping global changes in nuclear cytosine base modifications in the early mouse embryo. Reproduction.

[31] Hahn MA, Qiu R, Wu X, Li AX, Zhang H, Wang J (2013). Dynamics of 5-hydroxymethylcytosine and chromatin marks in mammalian neurogenesis. Cell Rep.

[32] Iurlaro M, Ficz G, Oxley D, Raiber EA, Bachman M, Booth MJ (2013). A screen for hydroxymethylcytosine and formylcytosine binding proteins suggests functions in transcription and chromatin regulation. Genome Biol.

[33] Park JS, Jeong YS, Shin ST, Lee KK, Kang YK (2007). Dynamic DNA methylation reprogramming: active demethylation and immediate remethylation in the male pronucleus of bovine zygotes. Dev Dyn.

[34] Abdalla H, Hirabayashi M, Hochi S (2009). Demethylation dynamics of the paternal genome in pronuclear-stage bovine zygotes produced by in vitro fertilization and ooplasmic injection of freeze-thawed or freeze-dried spermatozoa. J Reprod Dev.

[35] Galli C, Colleoni S, Duchi R, Lagutina I, Lazzari G (2007). Developmental competence of equine oocytes and embryos obtained by in vitro procedures ranging from in vitro maturation and ICSI to embryo culture, cryopreservation and somatic cell nuclear transfer. Anim Reprod Sci.

[36] Polanski Z, Motosugi N, Tsurumi C, Hiiragi T, Hoffmann S (2008). Hypomethylation of paternal DNA in the late mouse zygote is not essential for development. Int J Dev Biol.

[37] Smits K, Govaere J, Hoogewijs M, Piepers S, Van Soom A (2012). A pilot comparison of laser-assisted vs piezo drill ICSI for the in vitro production of horse embryos. Reprod Domest Anim.

[38] Galli C, Colleoni S, Duchi R, Lagutina I, Lazzari G (2007). Developmental competence of equine oocytes and embryos obtained by in vitro procedures ranging from in vitro maturation and ICSI to embryo culture, cryopreservation and somatic cell nuclear transfer. Anim Reprod Sci.

[39] Suzuki T, Fujikura K, Higashiyama T, Takata K (1997). DNA staining for fluorescence and laser confocal microscopy. J Histochem Cytochem.

